# The high efficient catalytic properties for thermal decomposition of ammonium perchlorate using mesoporous ZnCo_2_O_4_ rods synthesized by oxalate co-precipitation method

**DOI:** 10.1038/s41598-018-26022-2

**Published:** 2018-05-15

**Authors:** Xuechun Xiao, Bingguo Peng, Linfeng Cai, Xuanming Zhang, Sirui Liu, Yude Wang

**Affiliations:** 1 School of Materials Science and Engineering, Yunnan University, 650091 Kunming, People’s Republic of China; 2 Department of Physics, Yunnan University, 650091 Kunming, People’s Republic of China

## Abstract

Mesoporous ZnCo_2_O_4_ rods have been successfully prepared via oxalate co-precipitation method without any template. The nano-sized spinel crystallites connected together to form mesoporous structure by annealing homogeneous complex oxalates precursor at a low rate of heating. It is found that the low anneal rate plays an important role for the formation of mesoporous ZnCo_2_O_4_ rods. The effects of the heat temperature on the phase, morphology and catalytic properties of the products were studied. The XRD, SEM TEM, and N_2_ absorption/desorption have been done to obtain compositional and morphological information as well as BET surface area of the as-prepared sample. Catalytic activities of mesoporous ZnCo_2_O_4_ rods toward the thermal decomposition of ammonium perchlorate (AP) were investigated with differential scanning calorimetry (DSC) and thermogravimetry (TG) techniques. The results show that the addition of ZnCo_2_O_4_ rods to AP dramatically reduces the decomposition temperature. The ZnCo_2_O_4_ rods annealed at 250 °C possesses much larger specific area and exhibits excellent catalytic activity (decrease the high decomposition temperature of AP by 162.2 °C). The obtained mesoporous ZnCo_2_O_4_ rods are promising as excellent catalyst for the thermal decomposition of AP.

## Introduction

Due to their various applications including propulsion for large space vehicles and tactical missiles, gas generators for airbags, composite solid propellants are being intensively pursued^1–3^. Ammonium perchlorate (AP), as the main high energy constituent of solid propellants in the national defense field, accounts for 60–90% of the total weight of composite propellants^4^. Hence, the burning velocity and energy features of the propellants are significantly affected by the decomposition of AP. A comprehensive research about the thermal decomposition of AP was carried out by researchers^5^. Results indicate that a small quantity of catalysts can reduce the thermal decomposition temperature of AP, especially that corresponding to the high temperature decomposition (HTD), boosting up the apparent decomposition heat of AP, and increasing the burning velocity and efficiency of propellant accordingly. Recently, various kinds of transition metal oxides have been explored as catalyst for use in the thermal decomposition of AP, primarily including Fe_2_O_3_, CuO, Co_3_O_4_, NiO, ZnO and other metal oxide powders^6–9^. Compared to the single metal oxide, the complex oxides (containing two or more types of cations) with spinel structure have allured a lot of attention in material research because that the stabilization of active phases and synergistic interactions between two different oxides may improve its catalytic performance^10–12^. The presence of a partially filled-3d orbital in CuCo_2_O_4_ structure, so easy to accept electrons and improve the transferring electrons from perchlorate ions to the ammonium ions, which exhibits its stronger catalytic activity than probably CuO and Co_3_O_4_ as reported by Gheshlaghi *et al*.^13^. On account of the synergistic effect between Cu^2+^ and Cr^3+^ in the CuCr_2_O_4_ nanoparticles, the CuCr_2_O_4_ exhibits better catalytic effect than CuO^14,15^.

As a binary oxide, ZnCo_2_O_4_ is an attractive material with the bivalent Zn-ions occupying the tetrahedral sites in the cubic spinel structure and the trivalent Co-ions occupying the octahedral sites. ZnCo_2_O_4_ has recently attracted more and more attention for applying in catalyst in thermal decomposition of AP, owing to its excellent physicochemical performance and its abundant resources, low cost and environmental friendliness^16,17^. Porous structured materials have gained great attention for their superior physicochemical characters, including large specific area, high porosity, low density, high permeability and high adsorption performance. Hence, reports are available on the application of this type of materials in catalyst, for the fact that it favors the exposure of active sites and offer rapid mass transfer processes^18,19^. So it is an important part of this article to make further improvement on the catalytic activities of AP by introducing porous structure into spinel ZnCo_2_O_4_. Up to now, most approaches towards porous materials focus on template-assisted processes, including hard templates (porous silicon and polystyrene sphere) and soft templates (surfactants and block copolymers). Tomboc *et al*. synthesized hierarchical mesoporous ZnCo_2_O_4_ via using PVP as the soft template^20^. Highly ordered mesoporous spinel ZnCo_2_O_4_ with high surface area and narrow pore size was synthesized by using SBA-15 as the hard templates^21^. However, this synthetic route involves multi-step process, which is time-consuming and relatively complicated. So, it is very necessary to look for a simple and effective method to synthesize porous structure ZnCo_2_O_4_. A facial oxalate co-precipitation method to fabricate mesoporous spinel Co_3_O_4_ and MCo_2_O_4(4.5)_ (M = Mn, Ni, Fe, Cu) with high surface areas by a controlled pyrolytic of metal oxalate precursor as reported in^19,22^. This strategy is simpler and more efficient compared with other porous-casting method.

In this paper, the mesoporous ZnCo_2_O_4_ rod has been successfully synthesized via oxalate co-precipitation method without any template. The nano-sized spinel crystallites connected together to form mesoporous structure by annealing homogeneous complex oxalates precursor at a low rate of heating. The effects of the heat temperature on the phase, morphology and catalytic properties of the products were studied. The composition, morphology, porous structure, surface area and the catalytic activities to AP’s thermal decomposition of as-prepared mesoporous ZnCo_2_O_4_ rod are investigated in detail. The preliminary analysis of catalytic mechanism is discussed.

## Experimental

All the reagents used in the experiments were purchased from commercial sources of analytical grade and used without further purification.

### Preparation of mesoporous ZnCo_2_O_4_ rod

The typical procedure adopted for the preparation of mesoporous ZnCo_2_O_4_ rod is as follows: 16 mM Co(NO_3_)_2_·6H_2_O and 8 mM Zn(NO_3_)_2_·6H_2_O were successively dissolved in 80 mL deionized water followed by magnetic stirring for 30 min to obtain a homogeneous solution. Subsequently, the 24 mL saturated sodium oxalate solutions was added slowly. After continuous stirring for 2 h, the precipitate was collected by centrifugation and washed with deionized water and absolute ethanol several times and dried in air at 60 °C overnight.

Afterward, the pink precursor was annealed at various temperature (250 °C, 300 °C, 350 °C and 400 °C) for 2 h at a lower heating rate (1 °C/min) in temperature programming furnace. After that, the product was cooled inside the furnace to room temperature. The resultant black powder was collected and directly subjected to the various characterizations.

### Characterization of mesoporous ZnMCo_2_O_4_ rod

X-ray powder diffraction (XRD) patterns of the product was carried out on a Rigaku D/max-3B diffractometer with an incident X-ray wavelength of 1.540 Å (Cu Kα line), operated at 40 kV, 100 mA. The morphology was observed using field emission scanning electron microscopy (FESEM) taken on FEI nova nanosem 450 with microscope operating at 30 kV. Detailed studies of the microstructure were also carried out by transmission electron microscopy (TEM) (JEOL JEM-2100) at an acceleration voltage of 200 kV. XPS was carried out at room temperature in a PHI 5500 spectrometer with polychromatic Al/Mg-Kα X-ray source. During XPS analysis, Al K*α* X-ray beam was adopted as the excitation source and power was set to 250 W. Vacuum pressure of the instrument chamber was 1 × 10^−7^ Pa as read on the panel. Measured spectra were decomposed into Gaussian components by a least-square fitting method. Bonding energy was calibrated with reference to C1s peak (285.0 eV). The pore size distributions and the BET surface areas were measured by nitrogen adsorption/desorption using a NOVA2200e gas sorption analyzer (Quantachrome Corp.). Prior to the measurements, the sample was degassed at 300 °C in vacuum for 3 h. The functional groups and coordination of the samples were studied via the FT-IR analysis performed on the Nicolet iS 10 (Semerel technology Corp.), the frequency is range from 4000 to 400 cm^−1^. Thermogravimetric and differential thermal analysis (TG-DTA) were performed on a HCT-3 thermal analyzer (Bei-jing) at a heating rate of 10 °C/min from 25 °C to 400 °C.

### Catalytic performance to thermal decomposition of AP

The as-prepared sample was mixed with AP to reach certain mass ratios of 2%, 5%, 7% and 10%, respectively. The mixture was fully grinded in the presence of a certain amount of anhydrous ethanol until the ethanol volatilize. Afterwards, the catalytic activities of mesoporous ZnCo_2_O_4_ rod in the thermal decomposition of AP were performed using a HCT-3 thermal analyzer (Bei-jing) at a heating rate of 20 °C/min in nitrogen atmosphere over the range of 25–500 °C.

## Results and Discussion

**Figure 1:**
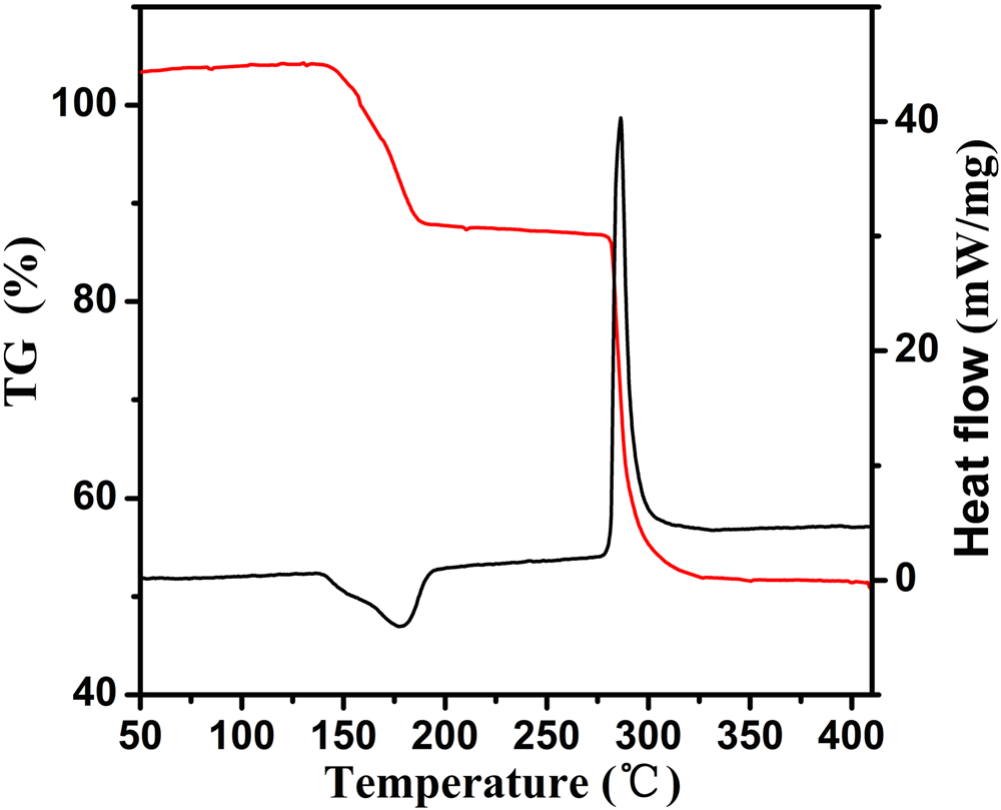
The TG and DTA curves for as-prepared precursor.

Researches show that the pyrolytic process of precipitated precursors has a strong effect on the crystallite phase and the morphology features (crystallite size, surface area, etc.) of the product^23^. Therefore, the pyrolytic behavior of homogeneous complex oxalates precursor was first explored by TG-DTA analysis in air atmosphere, the results of which is showed in Fig. 1. From the DTA curve, it can be seen that the endothermic process (the coordinated water elimination) happens at about 177 °C, and the precipitated precursor lost 12% of its original weight in the endothermic step as revealed by the TG curve. With increasing temperature, there is a rapid decline in mass (approximately 48% of its original) shown in the TG curve, which indicates the decomposition of oxalic groups and oxidation of the precursor into crystalline ZnCo_2_O_4_. This transitional process is accompanied by a strong exothermic peak at approximately 285 °C in the DTA curve. The single-phase feature of cobalt-zinc complex oxalate is further confirmed by the phenomenon that only one dehydration and one decomposition stage can be observed, as the endothermic and exothermic peaks of zinc oxalate do not accord with those of cobalt. Thus, it can be concluded that the zinc and cobalt atoms distribute on a molecular level in the resulting oxide lattice^19^. The decomposition temperature has huge effect on the crystalline size and the surface area of the resulting sample, and the anneal temperature can be reduced finely with the heating rate decreasing^22,23^. Hence, in this paper, in order to unveil the effects of the heat temperature on the phase, morphology and catalytic properties of the products, the pyrolysis temperature was set at various values from 250 °C to 400 °C on the basis of the above TG-DTA analysis.

**Figure 2:**
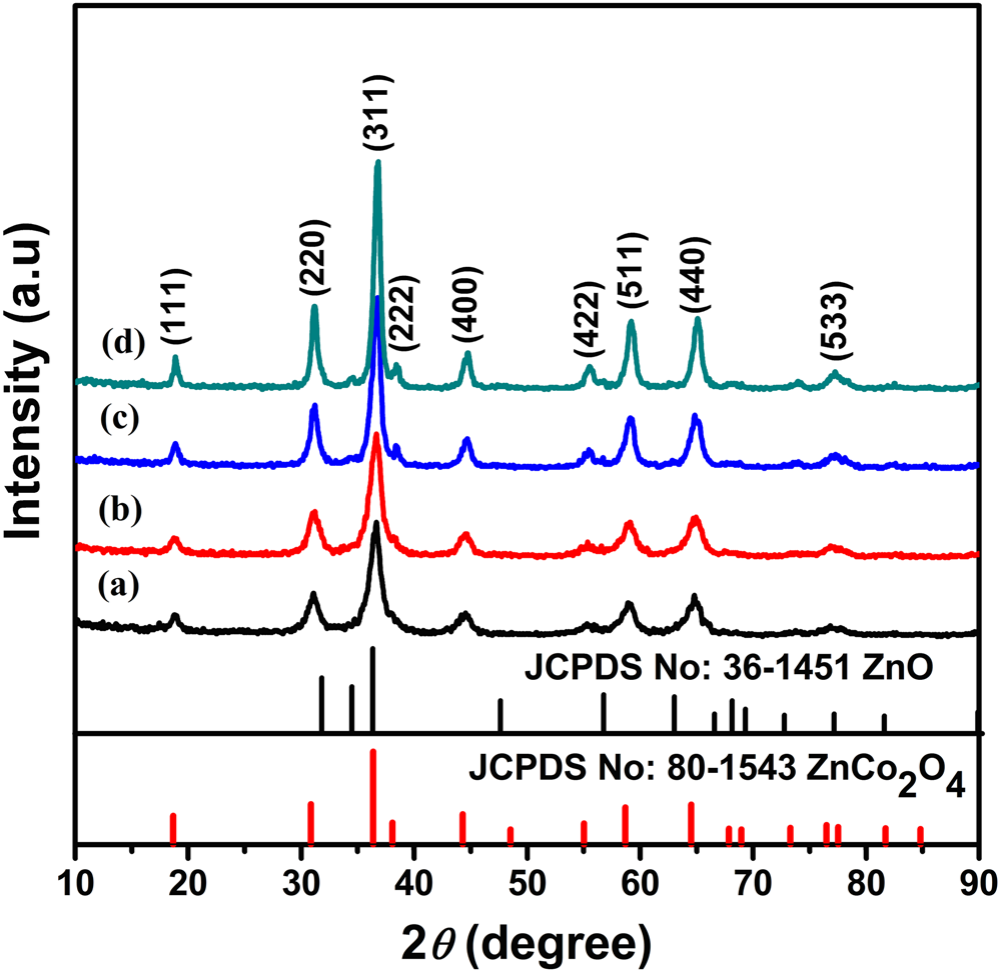
The XRD pattern of the ZnCo_2_O_4_ at different calcined temperature: (**a**) 250 ºC, (**b**) 300 ºC, (**c**) 350 ºC and (**d**) 400 ºC, respectively.

XRD patterns of final products obtained by sintering the precursor at 250 °C, 300 °C, 350 °C and 400 °C are shown in Fig. 2(a–d) correspondingly. All the diffraction peaks of the samples are in good consistency with the that of spinel ZnCo_2_O_4_ (JCPDS card no. 80-1543). It also can be seen that the diffraction peaks become increasingly sharp as the calcination temperature elevating, which confirms better crystallization and bigger grain size of the decomposed products with the temperature elevating. But there are diffraction peaks originating from the other phases besides the pure ZnCo_2_O_4_ phase (curve (c,d) in Fig. 2) in the XRD spectrum when the heat temperature increases. The peak positions of the additional phases agree with the standard values of the wurtzite ZnO (JCPDS No. 36-1451).

**Figure 3:**
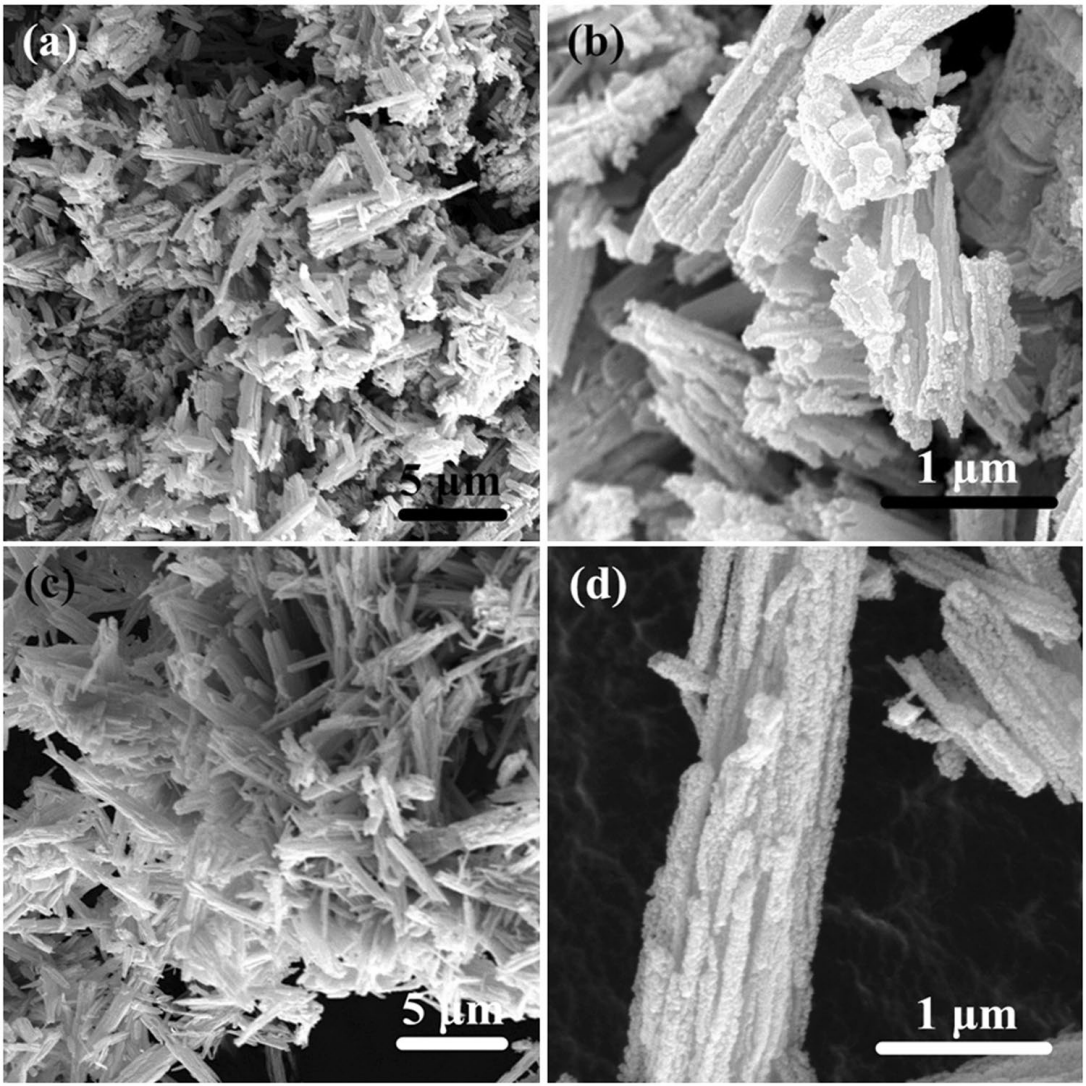
FESEM images of the ZnCo_2_O_4_ at different calcined temperature: (**a**) and (**b**) 250 ºC, (**c**) and (**b**) 400 ºC, respectively

Surface morphologies of the as-synthesized ZnCo_2_O_4_ calcined at 250 °C and 400 °C are studied with FESEM, and the obtained results are shown in Fig. 3. Figure 3(a,b) reveals the LRSEM and HRSEM images of the product calcined at 250 °C, and Fig. 3(c,d) corresponds to 400 °C. From the Fig. 3, it can be seen that the morphology of the pyrolysis products are nano-sized crystallites connected together to form mesoporous rod structure, and no obvious changes of the integral structure are observed as the heating temperature increases.

**Figure 4:**
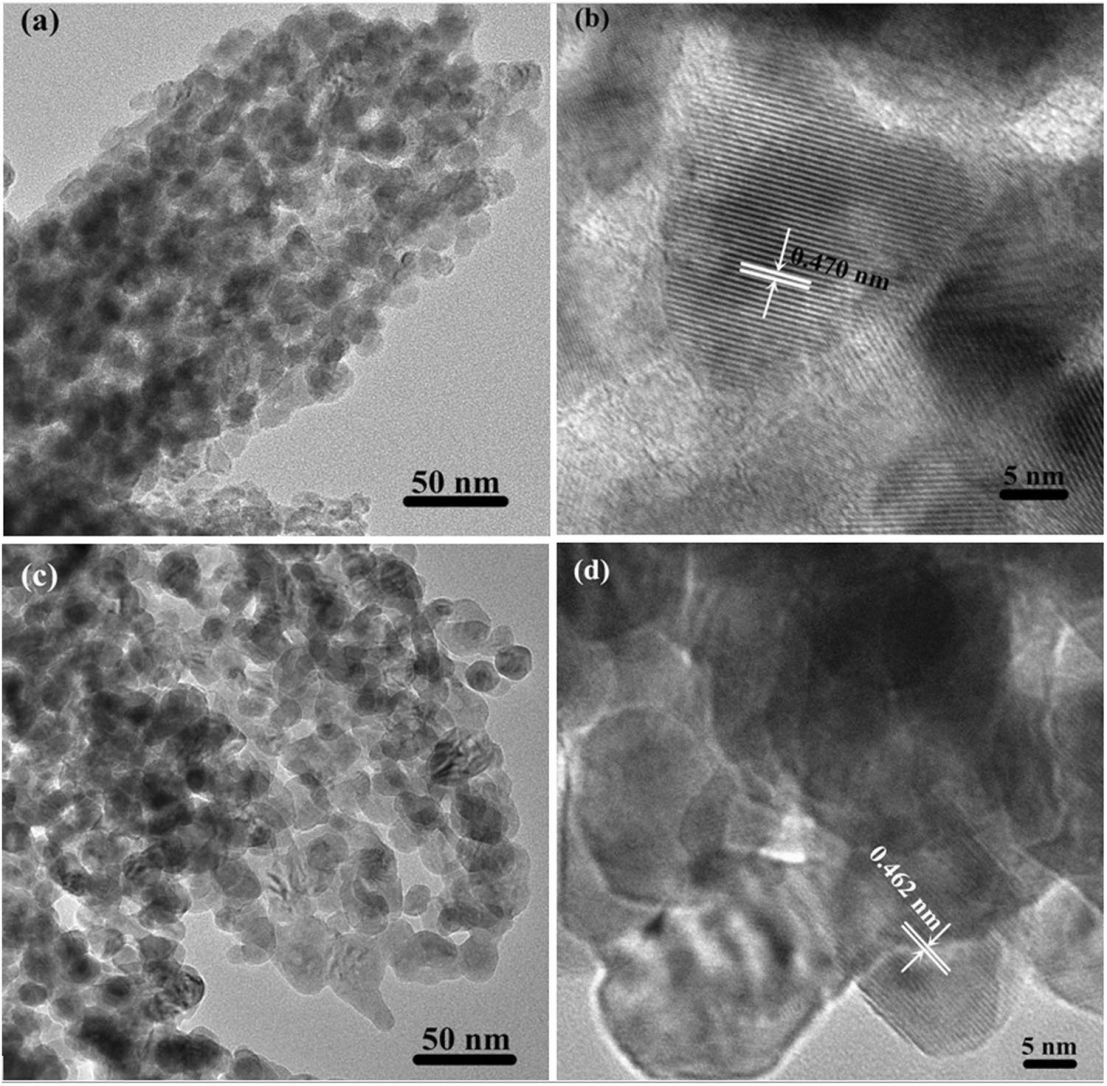
TEM and HRTEM images of the ZnCo_2_O_4_ at different calcined temperature: (**a**) and (**b**) 250 ºC, (**c**) and (**b**) 400 ºC, respectively.

The more information about the shape and crystallite size of the calcined products were further analysed by TEM (Fig. 4). Figure 4(a,b) display the TEM images of the sample calcined at 250 °C with low and high magnification, respectively. From these images it can be seen that the as-synthesized ZnCo_2_O_4_ nano-crystals interconnected together to form porous structure. The low and high magnification TEM images of the ZnCo_2_O_4_ annealed at 400 °C are exhibited in Fig. 4(c,d). These images demonstrate that the overall porous structure did not change at all, while enlargement of the crystalline size can be observed. In addition, lattice spacing between adjacent planes measured in the high-resolution TEM images (Fig. 4(b,d)) is 0.470 nm, and 0.462 nm, showing agreements with the distance between (111) crystal planes of cubic spinel ZnCo_2_O_4_. The porous structure might be formed by large amounts of gases slowly released from the micrometer sized oxalate particles leaving over plenty of space during the pyrolysis process^19^.

**Figure 5:**
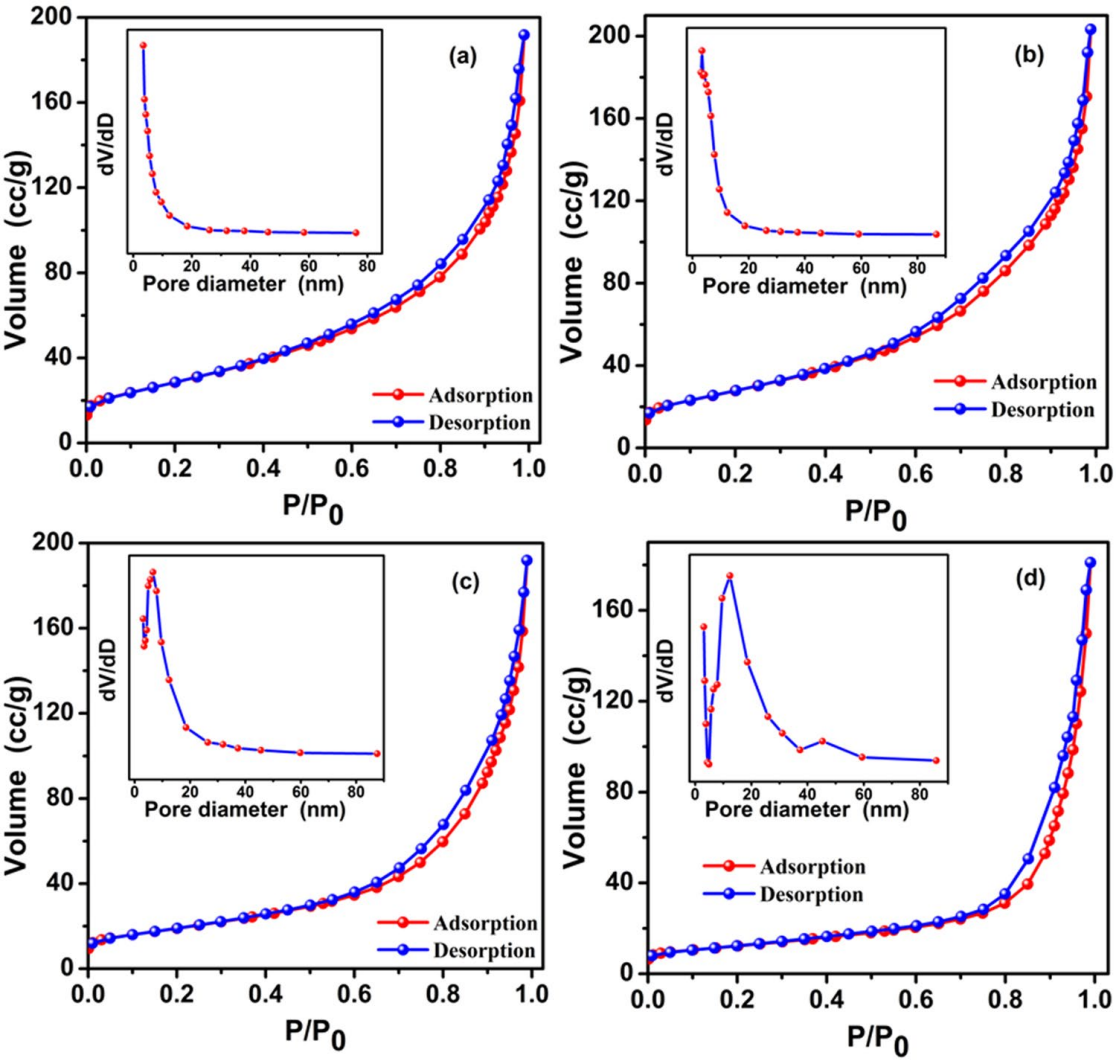
Nitrogen adsorption-desorption isotherm of ZnCo_2_O_4_ at different calcined temperature: (**a**) 250 ºC, (**b**) 300 ºC, (**c**) 350 ºC and (**d**) 400 ºC, respectively. Insert is the pore-size distribution calculated by the BJH method from the desorption branch of the ZnCo_2_O_4_.

The pore size distribution and surface area of the as-synthesized ZnCo_2_O_4_ obtained at different calcination temperature were measured by nitrogen adsorption/desorption method at 77 K. Figure 5 are N_2_ adsorption-desorption isotherms and corresponding pore size distribution plots (plotted by using the BJH calculation model) of the as-synthesized ZnCo_2_O_4_. As can be seen in Fig. 5, all of the porous ZnCo_2_O_4_ calcined at different temperatures exhibit a type IV isotherms and H3 hysteresis loop according to the IUPAC classification, which suggest that the mesoporous structure is formed by slit-like mesopore. Those pores were produced form the decomposition of cobalt - zinc oxalate hydrate crystallization during the pyrolysis process. The structural parameters and BET specific surface areas of the as-prepared ZnCo_2_O_4_ are derived from the isotherms and tabulated in Table 1. The BET surface areas of ZnCo_2_O_4_ calcined at 250 °C, 300 °C, 350 °C, 400 °C are determined to be 102.34, 101.36, 68.02 and 43.25 m^2^·g^−1^, respectively. The above data show that the calcination temperature has a significant impact on the specific surface areas of the products, i.e., the high calcination temperature will induce extensive growth of the spinel ZnCo_2_O_4_ and the collapse of the pore network. Thus, the specific surface area of ZnCo_2_O_4_ calcined at 400 °C dropped to 43.25 m^2^·g^−1^. From the pore size distribution curve (embedding figure in Fig. 5), it can be seen the size of pores exhibits a strong peak between 2.0 nm to 50 nm for the ZnCo_2_O_4_ calcined at different temperature, further verifying the presence of the mesoporous structure in the as-synthesized ZnCo_2_O_4_. The BJH pore volume of ZnCo_2_O_4_ calcined at 250 °C, 300 °C, 350 °C, 400 °C is 0.256, 0.294, 0.275 and 0.261 cm^3^·g^−1^ based on the calculation, respectively.

**Table 1:** The structural parameters and BET specific surface area of ZnCo_2_O_4_ at different calcined temperature.

Samples	BJH pore size (nm)	BJH pore volume (cm^3^/g)	BET specific surface area (m^2^/g)
ZnCo_2_O_4_ (250 °C)	3.410	0.256	102.335
ZnCo_2_O_4_ (300 °C)	5.618	0.294	101.358
ZnCo_2_O_4_ (350 °C)	7.829	0.275	68.021
ZnCo_2_O_4_ (400 °C)	12.379	0.261	43.247

**Figure 6:**
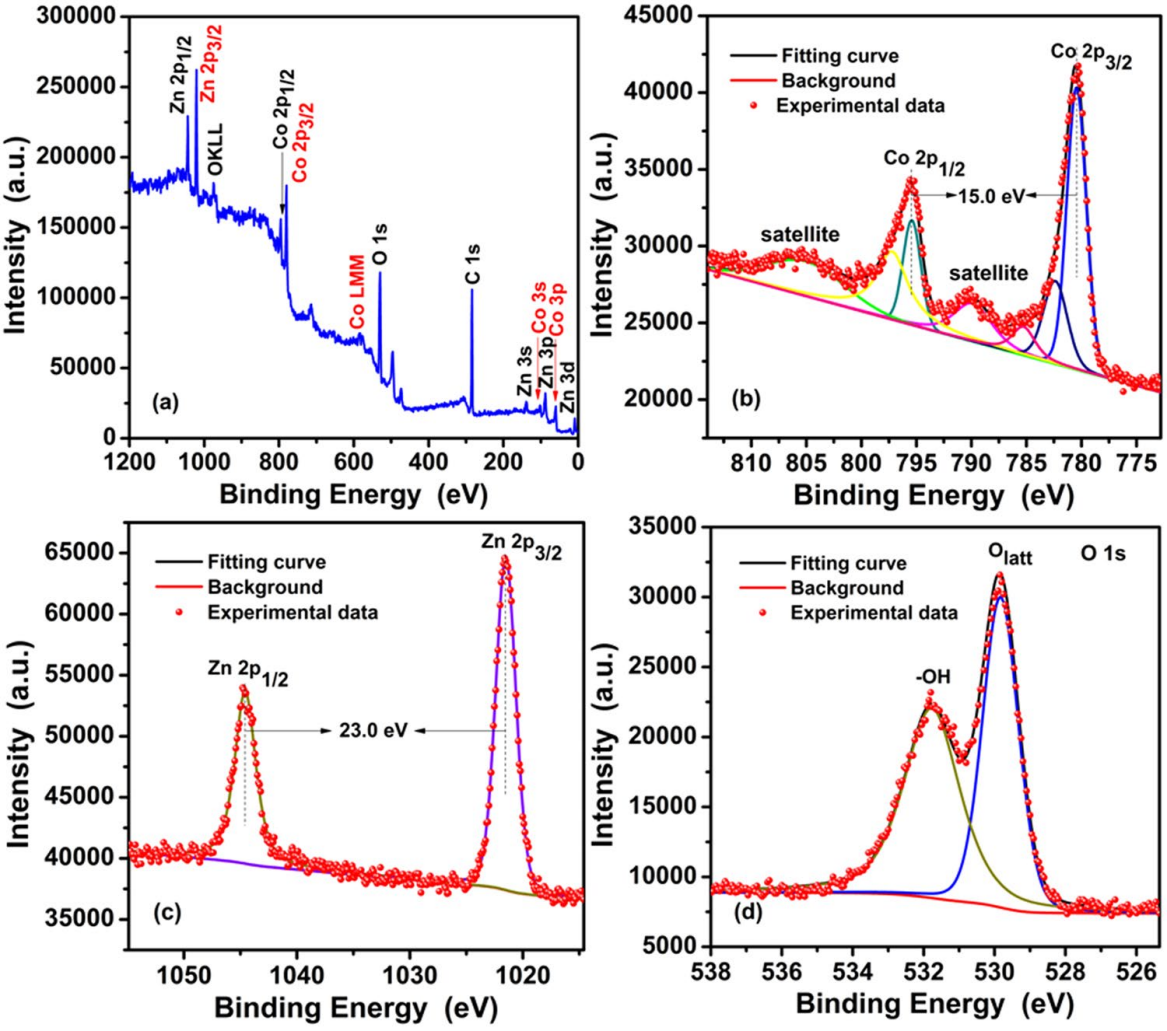
XPS spectra: (**a**) survey spectrum, (**b**) Co 2p, (**c**) Zn 2p, and (**d**) O 1s for the as-synthesized ZnCo_2_O_4_ calcined at 250 ºC.

The chemical states and surface properties of as-synthesized ZnCo_2_O_4_ calcined at 250 °C were analyzed via X-ray photoelectron spectroscopy (XPS), as shown in Fig. 6. Figure 6(a) shows a full survey spectrum of the ZnCo_2_O_4_ rod. Characteristic peaks for Zn, Co, O and C elements can be observed in the obtained curve. The binding energy values of the major peaks are 780.5 and 795.5 eV in the Co 2p spectrum (Fig. 6(b)), corresponding to Co 2p3/2 and Co 2p1/2, respectively. Additionally, the spine-orbit splitting of the mentioned two peaks is 15.0 eV, in accordance with data reported in the literatures^24,25^. Two accompanied weak satellite peaks located at 790.1 and 805.4 eV can also be observed, and the energy gap between the main peak and the satellite peaks is around 9.6 eV. This suggests that Co cation should be trivalent^26^. The strong resolution Zn 2p spectrum is presented in (Fig. 6(c)), in which two strong peaks at 1021.50 and 1044.50 eV can be clearly seen, corresponding to the binding energy of Zn 2p_3/2_ and Zn 2p_1/2,_ respectively, indicating the presence of Zn^2+^ in the ZnCo_2_O_4_ structure. It is observed that there is an energy separation of 23 eV between the Zn 2p_3/2_ and Zn 2p_1/2_ peaks, which is in agreement with an earlier report on ZnCo_2_O_4_^27^. From the O1s spectrum (Fig. 6(d)), it can be seen that the spectrum can be fitted to two gauss peaks at 529.8 eV and 531.7 eV are attributed to the lattice oxygen from the ZnCo_2_O_4_ rod and the oxygen from hydroxide ions^28^.

**Figure 7:**
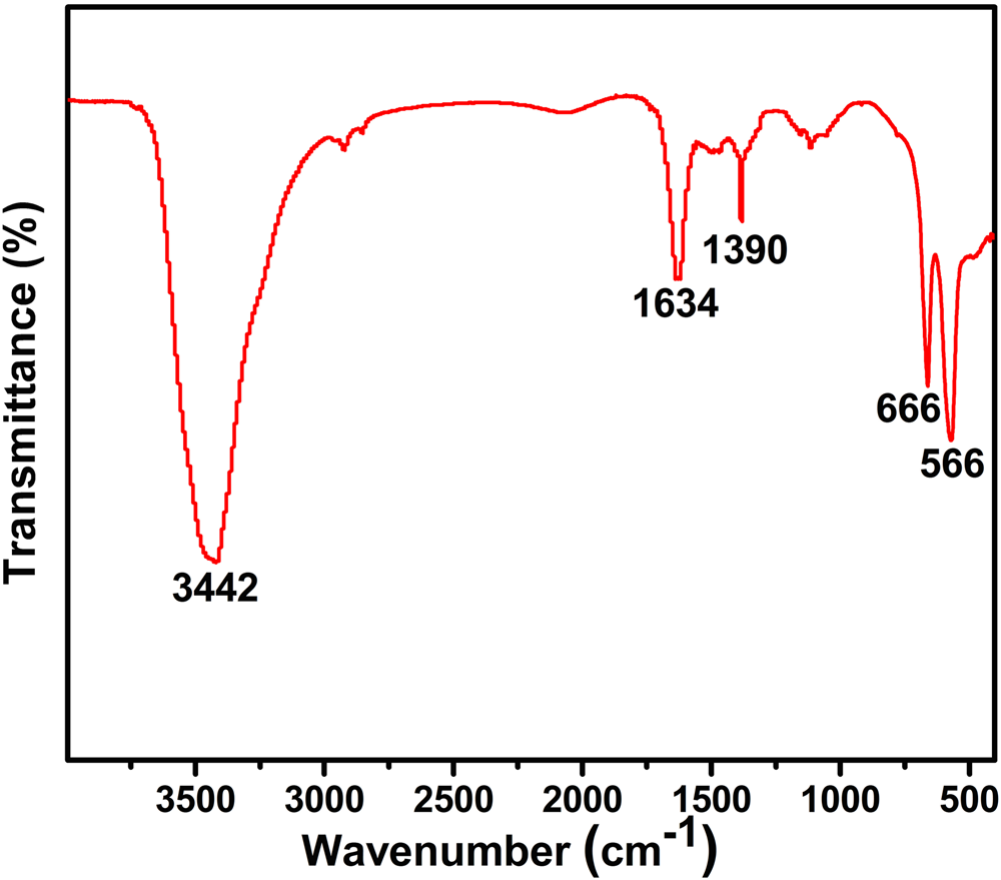
Typical FT-IR spectrum of the as-synthesized ZnCo_2_O_4_ calcined at 250 ºC.

The FT-IR spectrum of the as-synthesized ZnCo_2_O_4_ rod calcined at 250 °C was displayed in the Fig. 7. The characteristic band around 3442 cm^−1^ is assigned to the stretching vibration mode of H-O-H group, indicating the presence of chemisorbed water molecules. The prominent band of CO_3_^2−^ ions at 1634 cm^−1^ and the symmetric vibration *v*_sym_ (COO^-^) at 1390 cm^−1^ were observed^29^. The bands at 666 and 566 cm^−1^ can be assigned to the metal-oxygen vibration frequency of the metal at tetrahedral clearance (Zn-O) and octahedral clearance (Co-O), indicating the formation of ZnCo_2_O_4_ spinel structure^30^.

The propellant’s burning rate is affected by the AP particle size^31,32^, the particle size of AP is studied through SEM before the thermal decomposition analysis. From the SEM images of pure AP (Fig. S1.), the AP was inhomogeneous bulk structure, and its size was micron level, from tens of micron to few hundreds micron.

**Figure 8:**
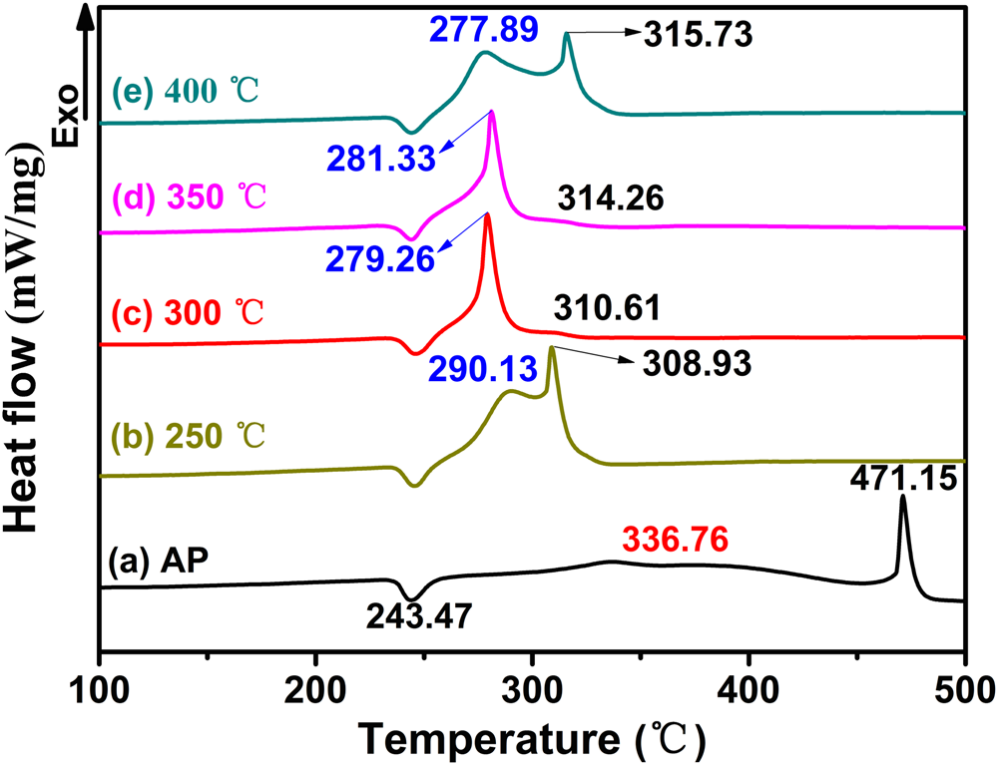
DSC curves of the AP decomposition in the absence and presence of ZnCo_2_O_4_ rod at different calcined temperature at a 2% mass basis: (**a**) pure AP, (**b**) 250 ºC, (**c**) 300 ºC, (**d**) 350 ºC and (**e**) 400 ºC, respectively.

**Figure 9:**
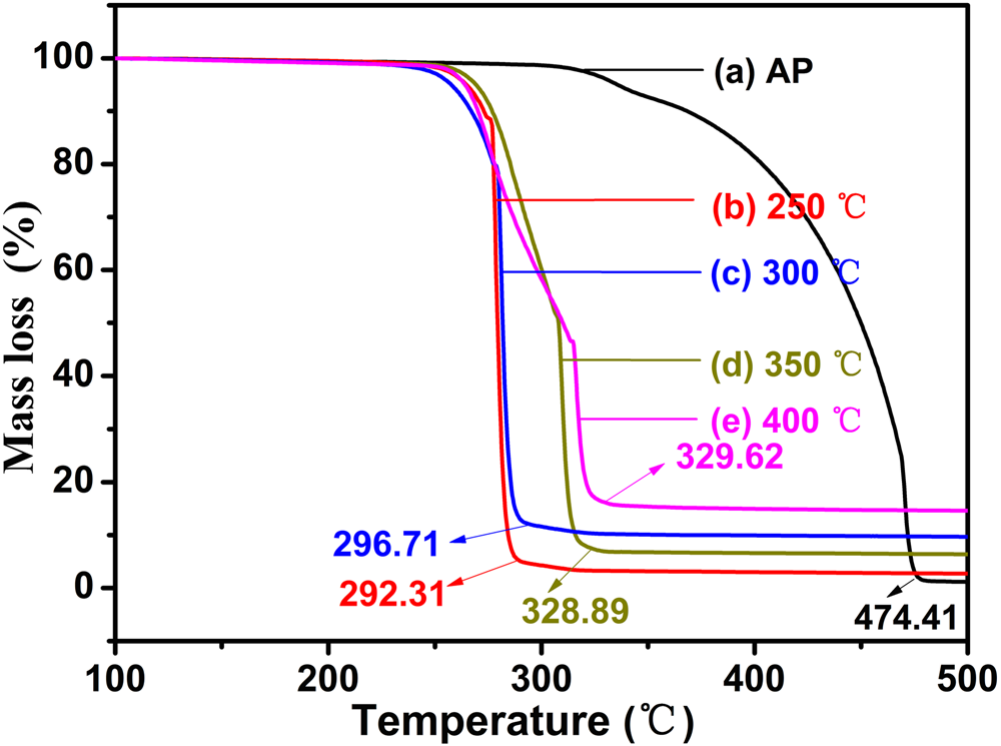
TG curves of the AP decomposition in the absence and presence of ZnCo_2_O_4_ rod at different calcined temperature at a 2% mass basis: (**a**) pure AP, (**b**) 250 ºC, (**c**) 300 ºC, (**d**) 350 ºC and (**e**) 400 ºC, respectively.

The catalytic performance of as-prepared ZnCo_2_O_4_ in the thermal decomposition of AP is demonstrated by the DSC analysis. The curves of AP decomposition in the absence and presence of ZnCo_2_O_4_ rod calcined at different temperature at a 2% mass basis are shown in Fig. 8. For pure AP (Fig. 8(a)), the curve indicates that the decomposition process of AP consists of three stages. In first stage, the endothermic peak demonstrates that AP undergoes a crystallographic transition from orthorhombic to cubic phase at 243.47 °C^33^, In the subsequent two stages, the following partial decomposition of AP at 336.76 °C is revealed by the low temperature decomposition (LTD) peak. After that, complete decomposition at 471.15 °C is revealed by the high temperature decomposition (HTD) peak^13^. Compared with the pure AP, an obvious difference for AP decomposition in the presence of mesoporous ZnCo_2_O_4_ rod with a mass ratio of 2% can be seen in Fig. 8. All of curves show endothermic peak at the almost same temperature, which indicates that the crystallographic phase transition hasn’t been affected by the addition of the mesoporous ZnCo_2_O_4_ rod, while significant declines in the value of temperature can be seen from LTD and HTD peaks (Fig. 8(b–e)). All of the HTD peaks of AP in the presence of the mesoporous ZnCo_2_O_4_ rod shifted even to the front of the LTD peaks of pure AP, indicating that the mesoporous ZnCo_2_O_4_ rod can immensely promote the thermal decomposition of AP. The specific thermal decomposition temperature data are summarized in Table 2. The HTD peaks of AP containing the 2% mesoporous ZnCo_2_O_4_ rod calcined at 250, 300, 350, and 400 °C as catalyst are 308.93, 310.61, 314.26 and 315.73 °C, respectively. These data show a decrease of 162.22, 160.54, 156.89 and 155.42 °C with respect to the pure AP. The results endorse the best catalytic performance of mesoporous ZnCo_2_O_4_ rod calcined at 250 °C. It is reasonable to propose that the relatively highest specific surface area and the lowest pore size of ZnCo_2_O_4_ rod calcined at 250 °C might be the origin of its extraordinary performance, as those paramaters are all crucial factors affecting of catalytic efficiency. From the TG curves (Fig. 9), it can be seen that within the scope of 100 °C to 500 °C, two weight loss steps are clearly observed for pure AP. The first weight loss can be attributed to the partial decomposition of AP and formation of some intermediates by dissociation and sublimation. The second weight loss is caused by the complete decomposition of the intermediate to volatile products^34^. Whereas only one weight loss presents in the decomposition of AP with 2% mesoporous ZnCo_2_O_4_ rod (w/w), indicating that the final decomposition temperature of AP had been significantly decreased by the additive mesoporous ZnCo_2_O_4_ rod calcined at different temperature (pure AP: 474.41 °C, AP + 2%ZnCo_2_O_4_ (400 °C): 329.62 °C, AP + 2%ZnCo_2_O_4_ (350 °C): 328.89 °C, AP + 2%ZnCo_2_O_4_ (300 °C): 296.71 °C, AP + 2%ZnCo_2_O_4_ (250 °C): 292.71 °C). This phenomenon considerably concurs with the exothermic peaks of the DSC curves. It can be seen that the mesoporous ZnCo_2_O_4_ rod calcined at 250 °C manifests highest catalytic activity. In addition, the catalytic activity of the mesoporous ZnCo_2_O_4_ rod are the highest than those reported in literatures^5,13,17,35–39^, as shown in Table 3.

**Table 2:** Data of the AP decomposition in the absence and presence of ZnCo_2_O_4_ (2%) at different calcined temperature.

Samples	PT Peak (°C)	LTD Peak (°C)	HTD Peak (°C)
Pure AP	243.47	336.68	471.15
ZnCo_2_O_4_ (250 °C)	244.98	290.13	308.93
ZnCo_2_O_4_ (300 °C)	244.98	279.26	310.61
ZnCo_2_O_4_ (350 °C)	244.77	281.33	314.26
ZnCo_2_O_4_ (400 °C)	244.34	277.89	315.73

Notes: In this table, PT, LTD and HTD represent crystallographic transition endothermic peak temperature, low-temperature decomposition and high-temperature decomposition respectively.

**Table 3:** Comparison of Catalytic activity for Ammonium Perchlorate of various transition metal oxides.

Materials	M%	*β* °C/min	LTD Peak (°C)		Decrease of LTD Peak (°C)	HTD Peak (°C)		Decrease of HTD Peak (°C)	Ref.
			Pure AP	AP+ Sample		Pure AP	AP+ Sample		
Nanoparticles MnFe_2_O_4_	3	20	328.7	289.2	39.54	430.2	345.3	84.9	^5^
Nanoparticles CuCr_2_O_4_	2	20	331			467	369.9	97.1	^14^
Nanorod ZnCo_2_O_4_	2	5	316.7	274.5	42.2	449.2	290.9	158.3	^17^
Nanopowders CuCo_2_O_4_	3	No description	331.1	308.43	22.71	443.6	340.8	102.8	^13^
Nanoparticles CoFe_2_O_4_	2	20	326.2	—	—	433.2	320.4	112.8	^35^
Nanoporous CoFe_2_O_4_	2	5	315.7	—	—	413.9	356.0	57.9	^36^
Microspheres Fe_3_O_4_	2	20	317	—	—	456	376	80	^37^
Microspheres Co_3_O_4_	2	20	317	—	—	456	382	74	
Nanometer CuFe_2_O_4_	2	10	333.3	318.4	14.9	445	353.8	91.2	^38^
Nanoparticles Co_3_O_4_	2	10	325	—	—	453.1	336.2	116.9	^39^
Porous ZnCo_2_O_4_ (250 °C)	2	20	336.7	290.13	46.55	471.2	309.1	162.01	This work

**Notes:** In this table, M, *β*, LTD and HTD represent the blend ratio of catalysts, heating rate, low-temperature decomposition and high-temperature decomposition respectively.

**Figure 10:**
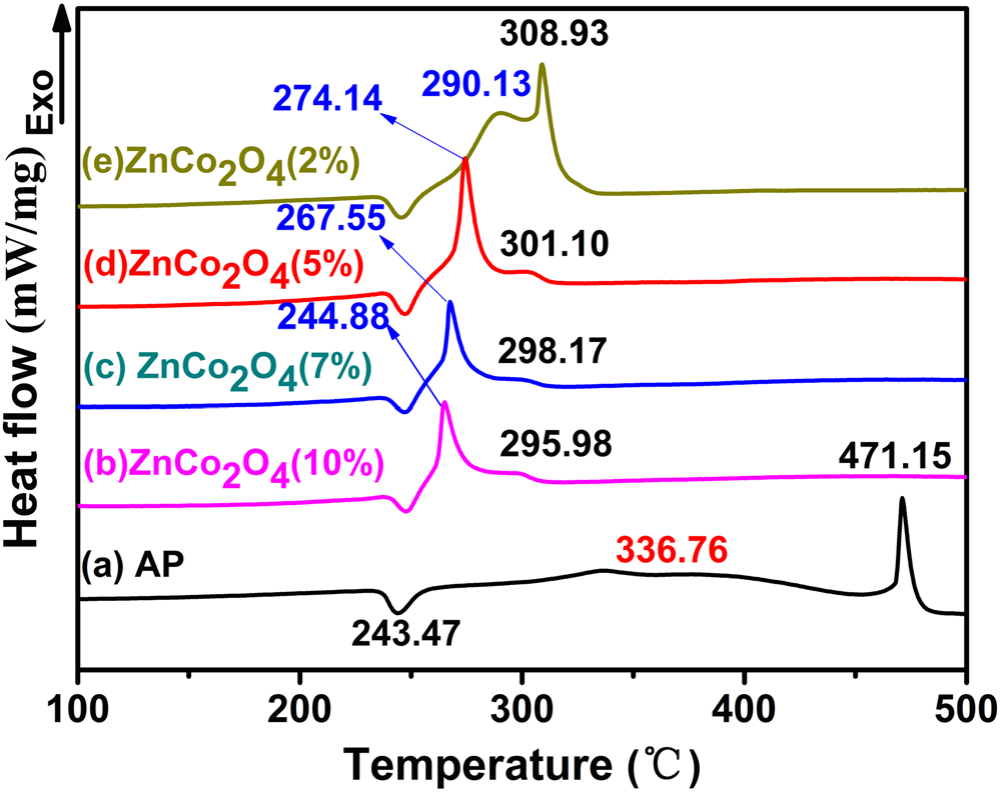
DSC curves of the AP decomposition in the presence of the ZnCo_2_O_4_ calcined at 250 ºC: (**a**) pure AP; (**b**) AP + ZnCo_2_O_4_(10%); (**c**) AP + ZnCo_2_O_4_(7%); (**d**) AP + ZnCo_2_O_4_(5%) (**e**) AP + ZnCo_2_O_4_(2%), respectively.

**Figure 11:**
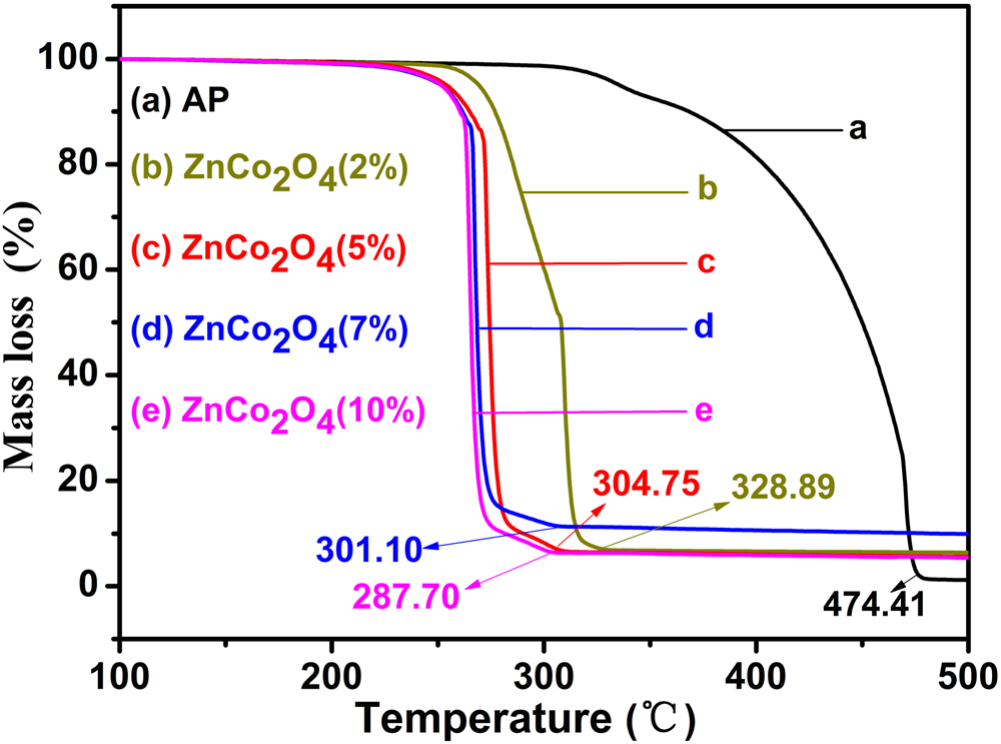
TG curves of the AP decomposition in the presence of the ZnCo_2_O_4_ calcined at 250 ºC: (**a**) pure AP; (**b**) AP + ZnCo_2_O_4_(2%); (**c**) AP + ZnCo_2_O_4_(5%); (**d**) AP + ZnCo_2_O_4_(7%) (**e**) AP + ZnCo_2_O_4_(10%), respectively.

The thermal decomposition of AP is also influenced by the blend ratio of mesoporous ZnCo_2_O_4_ rod. DSC curves of AP with a different mass ratio of mesoporous ZnCo_2_O_4_ rod calcined at 250 °C and pure AP are shown in Fig. 10. From Fig. 10, obvious changes can be observed in AP with the addition of mesoporous ZnCo_2_O_4_ rod (calcined at 250 °C) of different weight ratios. The HTD peaks of AP are lowered with the increasing mass ratio of the mesoporous ZnCo_2_O_4_ rod additive (from 308.93 °C at 2% mass ratio to 295.98 °C at 10% mass ratio). The results reveal that better catalytic performance is achieved with mesoporous ZnCo_2_O_4_ within the mixture. TG curves of pure AP and mixtures of ZnCo_2_O_4_ and AP with different mass ratios are given in Fig. 11. Two weight loss steps are present during the decomposition of AP, whereas only one step can be observed for mixtures according to the TG curves. Moreover, the temperature corresponding to the start of significant weight loss for mixtures decreases with increased the mass ratios. This finding considerably agrees with that derived from Fig. 10.

Up to now, the thermal decomposition mechanism of AP is not yet fully understood because the decomposition process of AP is a complex solid-gas multiphase reaction process involving reactions in the solid, absorbed and gaseous phases. Several unsolved issues remain till now^40,41^. At low temperatures decomposition, the AP decomposes leading to formation of a small number of intermediate products, the main pivotal step is that the electrons transfer from perchlorate ion to ammonium ion, which would transfer to NH_3_ and HClO_4_ by dissociation and sublimation and as follows:

1 $${{\rm{NH}}}_{4}{{\rm{ClO}}}_{4}\to {{{\rm{ClO}}}_{4}}^{-}+{{{\rm{NH}}}_{4}}^{+}\,\to \,{{{\rm{ClO}}}_{4}}^{0}+{{{\rm{NH}}}_{4}}^{0}\to {{\rm{HClO}}}_{4}+{{\rm{NH}}}_{3}$$

Boldyrev *et al*. assumed the electrons transfer to happen on the surface not interior of crystal^42^. After the NH_4_^+^ accepts electron to become activated NH_4_^0^, the activated ammonium radicals can decompose to ammonia and hydrogen atom:

2 $${{{\rm{NH}}}_{4}}^{0}\to {{\rm{NH}}}_{3}+{\rm{H}}$$

The activated ClO_4_^0^ can react with hydrogen atom and form HClO_4_, the HClO_4_ can further reacted with the H atom:

3 $${{\rm{HClO}}}_{4}+{\rm{H}}\to {{\rm{ClO}}}_{3}+{{\rm{H}}}_{2}{\rm{O}}$$

As an electron absorption body, ClO_3_ can converted into ClO_3_^−^ which then react with NH_3_ in the adsorbed gas to produce various such as NO, N_2_O, H_2_O^15^:

4 $${{\rm{ClO}}}_{3}+{{\rm{e}}}^{-}\to {{{\rm{ClO}}}_{3}}^{-}$$

The intermediate products NH_3_ and HClO_4_ by dissociation and sublimation of AP are not only absorbed on the surface of perchlorate crystal to react, but also desorbed and sublimed into the gas phase^35^. Because the adsorbed NH_3_ can not be completely oxidized by the decomposition products of HClO_4_ at low temperature, it overlays continually on the surface of AP. Hence, the NH_3_ adsorbed on the surface gets saturated, which causes cessation of the reaction and incomplete transformation of perchlorate. As the temperature rising sequentially, the reaction between NH_3_ and HClO_4_ absorbed on the surface of AP will be triggered again to produce final volatile products including HCl, H_2_O, NO, N_2_O and so on^43^. In the high-temperature decomposition process, the controlling step is the transformation from O_2_ to superoxide ion O_2_^−^, which can further react with NH_3_ to form N_2_O, NO_2_ and H_2_O^44^. Therefore, the high electron transfer capacity and large specific surface area of the catalyst have an important effect on the thermal decomposition of AP. According to the electron transfer mechanism, the ZnCo_2_O_4_ as p-type semiconductor materials, it has effective sites (positive holes on the surface of catalyst) to accept released electron from perchlorate, accompanied by the abstraction of atomic oxygen from the perchlorate ion^45^.

5 $${{{\rm{e}}}^{-}}_{{\rm{oxide}}}+{{{\rm{ClO}}}_{4}}^{-}\to {{\rm{O}}}_{{\rm{oxide}}}+{{{\rm{ClO}}}_{3}}^{-}\to 1/2{{\rm{O}}}_{2}+{{{\rm{e}}}^{-}}_{{\rm{oxide}}}+{{{\rm{ClO}}}_{3}}^{-}$$

where e^−^_oxide_ represents a positive hole in the valence band of the oxide and O_oxide_ is an abstracted oxygen atom from oxide. The mechanism of catalytic action is based on the presence of superoxide ion O_2_^−^ on the surface of the catalysts, so the ZnCo_2_O_4_ catalyst may promoted the dissociation of ClO_4_^−^ species into ClO_3_^−^ and O_2_. In addition, the transition metal ion Co^3+^ in the spinel structure of ZnCo_2_O_4_ has outermost d orbitals with 3d^6^ electronic configurations, and the d orbitals are not filled with electrons and have hole conductivity. It can easily accept the released electron from ClO_4_^−^ to form Co^2+^ (3d^7^) cations, the electron was transferred to the surface of the catalyst and reacted with NH_4_^+^ to decompose into ammonia and hydrogen atom^46^.

6 $${{\rm{Co}}}^{3+}+{{\rm{ClO}}}_{4}^{-}\to {{\rm{Co}}}^{2+}+{{\rm{ClO}}}_{4}^{0}$$

7 $${{\rm{Co}}}^{2+}+{{{\rm{NH}}}_{4}}^{+}\to {{\rm{Co}}}^{3+}+{{{\rm{NH}}}_{4}}^{0}$$

**Figure 12:**
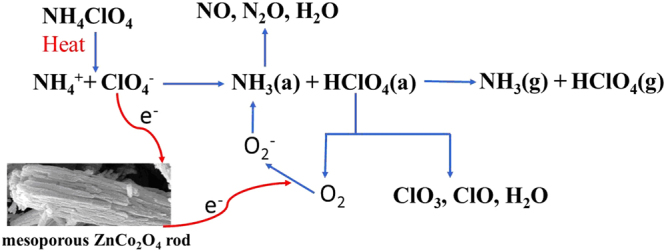
Schematic diagram of the thermal catalytic activity enhancement of mesoporous ZnCo_2_O_4_ rod.

**Figure 13:**
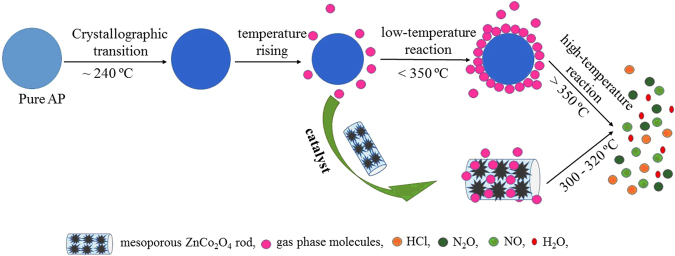
Flow diagram of the thermal decomposition process of AP in the absence and presence of mesoporous ZnCo_2_O_4_ rod.

In the thermal decomposition process of AP, ZnCo_2_O_4_ serves as a bridge for transferred electrons from perchlorate ions to the ammonium ions and the other transformation from O_2_ to superoxide ion O_2_^−^, as depicted in Fig. 12. In the end, positive synergistic catalytic effect of ternary oxide may also contribute to AP’s decomposition^13^. Due to the high specific surface area and great adsorption of the mesoporous ZnCo_2_O_4_ rods, the decomposed intermediate products in the gaseous phase of AP can be facilely adsorbed on the surface of mesoporous ZnCo_2_O_4_ rods shown in Fig. 13. Thus, the addition of mesoporous ZnCo_2_O_4_ to AP can increase the contact area of the catalytic reaction, augment the number of active sites, which promotes the thermal decomposition of AP. So that the mesoporous ZnCo_2_O_4_ rod calcined at 250 °C manifests highest catalytic activity than other products, which is mainly because of its relatively highest specific surface area.

## Conclusions

In summary, mesoporous ZnCo_2_O_4_ rod has been successfully synthesized via a controlled thermal decomposition of homogeneous complex oxalates precursor, which is no need of the assistance of soft/hard template. XRD, SEM, TEM, XPS and nitrogen adsorption/desorption have been done to systematically characterize the structural and morphological features of the as-prepared products. After calcined at given temperature with a low rate of heating, the nano-sized ZnCo_2_O_4_ crystallites connected together to form mesoporous rod. The as-prepared material ZnCo_2_O_4_ calcined at 250 °C showed much larger surface area (102.34 m^2^·g^−1^) and high catalytic activity, shifting the AP high thermal decomposition temperature downwardly to about 162.1 °C. The results suggest that the as-prepared mesoporous ZnCo_2_O_4_ rod has great catalytic properties on thermal decomposition of AP, which can be used as a promising additive in the future.

## Electronic supplementary material

Supplementary Information
