# Supplementary material for: The high efficient catalytic properties for thermal decomposition of ammonium perchlorate using mesoporous ZnCo2O4 rods synthesized by oxalate co-precipitation method
Source: Sci Rep. 2018 May 15;8:7571. doi: 10.1038/s41598-018-26022-2 (PMC5953971; doi:10.1038/s41598-018-26022-2)
Supplement: Supplementary file 1 — Supplementary Information [file 41598_2018_26022_MOESM1_ESM.doc]

Supplementary Information for

**The high efficient catalytic properties for thermal decomposition of ammonium perchlorate using mesoporous ZnCo_2_O_4_ rods synthesized by oxalate co-precipitation method**

Xuechun Xiao ^†,‡^, Bingguo Peng^†^, Linfeng cai^†^, Xuanming Zhang ^†^, Sirui Liu^†^, Yude Wang †,‡[[1]](#footnote-2)*

^†^ School of Materials Science and Engineering, Yunnan University, 650091 Kunming, People’s Republic of China

^‡^ Department of Physics, Yunnan University, 650091 Kunming, People’s Republic of China


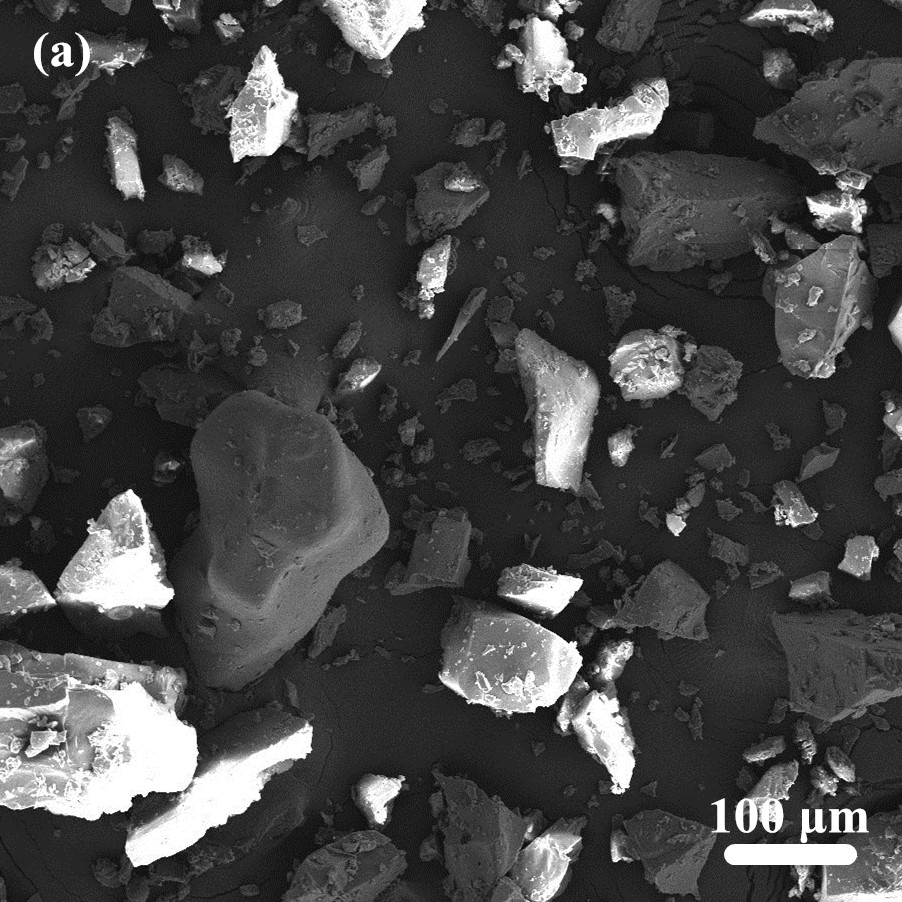

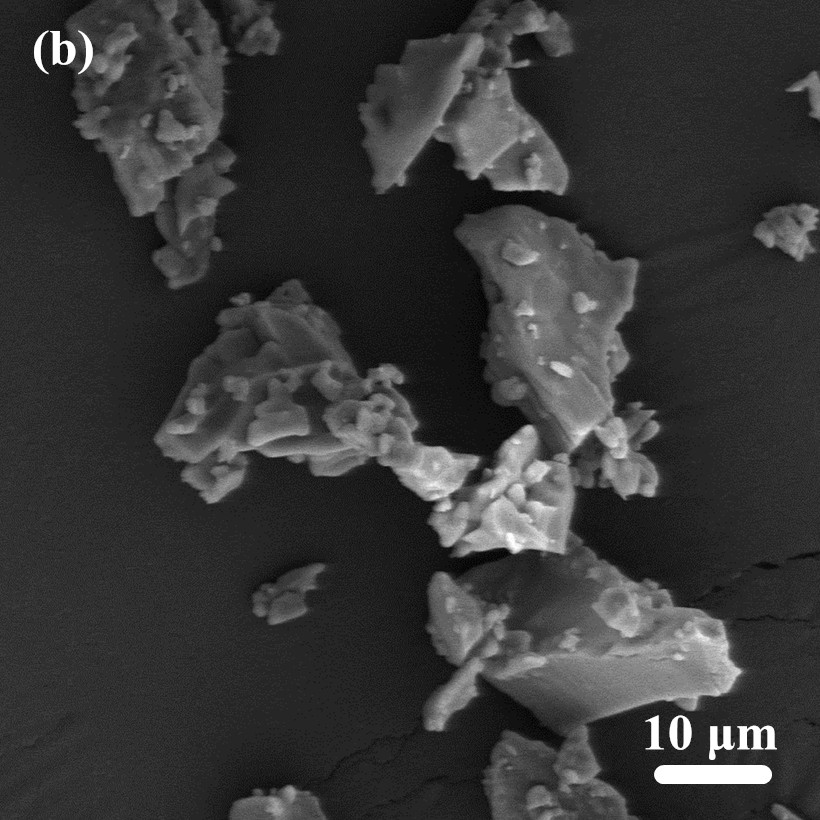


**Figure S1.** SEM images of the pure AP.

1. ^^ To whom correspondence should be addressed. Tel: +86-871-65035570, Fax: +86-871-65153832. E-mail: ydwang@ynu.edu.cn (Y. D. Wang) [↑](#footnote-ref-2)
